# De Novo Minimal Change Disease following Vaccination with the Pfizer/BioNTech SARS-CoV-2 Vaccine in a Living Kidney Donor

**DOI:** 10.3390/medicina58010037

**Published:** 2021-12-27

**Authors:** Smaragdi Marinaki, Kyriaki Kolovou, George Liapis, Chrysanthi Skalioti, Stathis Tsiakas, Ioannis Boletis

**Affiliations:** 1 Department of Nephrology and Renal Transplantation, Medical School, National and Kapodistrian University of Athens, Laiko Hospital, 11527 Athens, Greece; smaragdimarinaki@yahoo.com (S.M.); c_skalioti@yahoo.com (C.S.); stathis.tsiakas@gmail.com (S.T.); laikneph@laiko.gr (I.B.); 2 Department of Pathology, Laiko Hospital, 11527 Athens, Greece; gliapis@gmail.com

**Keywords:** COVID-19 vaccination, minimal change disease, kidney donor, de novo glomerulonephritis, SARS-CoV-2 mRNA vaccines

## Abstract

Coronavirus disease 2019 has developed as a pandemic. Immunization with the introduction of vaccines against COVID-19 seems be the only way to end this pandemic. We report on a case of a kidney donor, who developed minimal change disease (MCD) within 4 days post-vaccination with the SARS-CoV-2 BNT162b2 mRNA vaccine (Pfizer/BioNTech). She donated her kidney to her husband 4 years ago. After receiving the 1st vaccine dose, she presented with nephrotic syndrome, with complete remission 5 days later. She proceeded with the second dose of the BNT162b2 vaccine at the appointed time. Two days later, she presented with a relapse of full-blown nephrotic syndrome with preserved renal function. We performed an ultrasound-guided percutaneous kidney biopsy and the final diagnosis was consistent with minimal change disease. Oral prednisolone was promptly initiated at a dosage of 1 mg/kg daily and complete remission was achieved 10 days later. More data about this rare appearance of de novo glomerular diseases after SARS-CoV-2 vaccination are emerging and should be interpreted rigorously.

## 1. Introduction

Worldwide SARS-CoV-2 vaccination is the most powerful weapon against the COVID-19 pandemic. However, new reports of adverse events following mRNA-based Pfizer-BioNTech COVID-19 vaccine have emerged. We report a case of de novo MCD following vaccination against SARS-CoV-2 virus.

## 2. Case Presentation

A 55-year-old female kidney donor presented with pitting edema of the lower extremities. Four days earlier, she had received the first dose of the SARS-CoV-2 BNT162b2 mRNA vaccine (Pfizer/BioNTech).

The patient had donated her left kidney to her 69-year-old husband four years ago. She was on levothyroxine sodium 75 μcg daily due to hypothyroidism; her past medical history was otherwise unremarkable. Four months ago, during her regular follow-up appointment at the out-patient clinic, she presented with a creatinine level of 0.8 mg/dL and a urinary protein-to-creatinine ratio of 0.070 g/g and her blood pressure was within normal limits (at that time).

**Figure 1:**
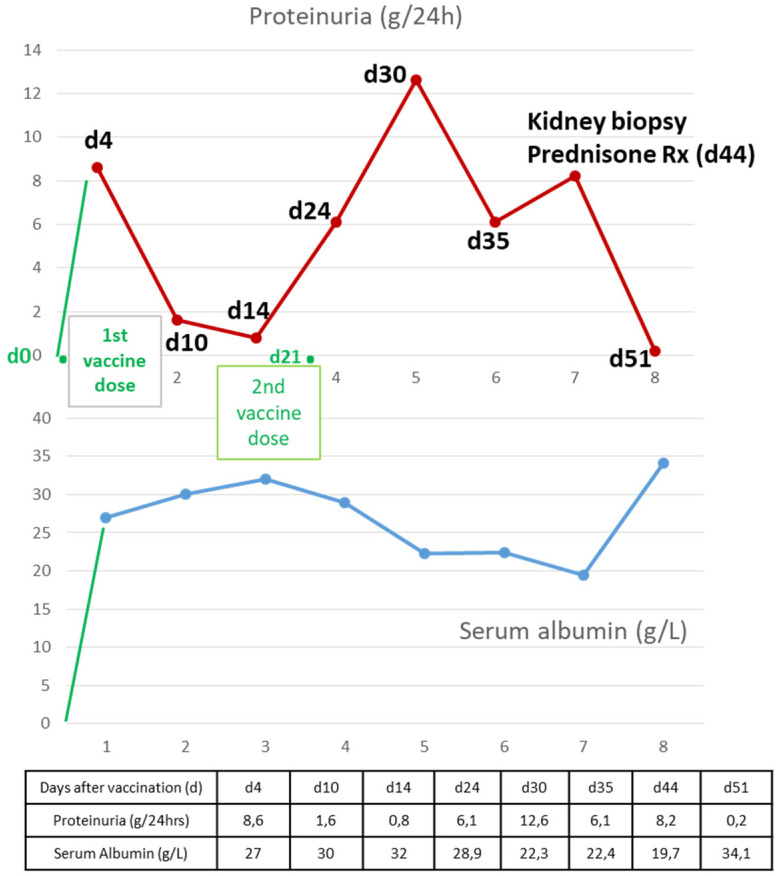
Laboratory data.

Four days post-vaccination with the 1st dose of the BNT162b2 vaccine, she noticed swelling of her lower limbs. At presentation, her laboratory tests revealed nephrotic syndrome with a urine protein level of 8.6 g/24 h, a serum albumin level of 27 g/L, hyperlipidemia, and normal renal function, with a creatinine level at her baseline value. No history of nonsteroidal anti-inflammatory drug use was reported. On physical examination, she had peripheral pitting edema, mild abdominal distension, and normal blood pressure. Loop diuretics were prescribed, and on her follow-up appointment 5 days later, physical examination and blood test results were indicative of disease remission; proteinuria had dropped to 1.6 g/24 h and serum albumin had increased to 30 g/L. Complete remission of nephrotic syndrome followed 5 days later (Figure 1).

She proceeded with the second dose of the BNT162b2 vaccine at the appointed time. Two days later, she presented with a relapse of full-blown nephrotic syndrome with preserved renal function. Due to anasarca worsening, with more than 15 kg of weight gain over the next few days, the patient was admitted to the hospital, where she was initially treated with intravenous bolus infusions of furosemide and human albumin. Furthermore, an ultrasound-guided percutaneous kidney biopsy was performed on her remnant right kidney.

**Figure 2:**
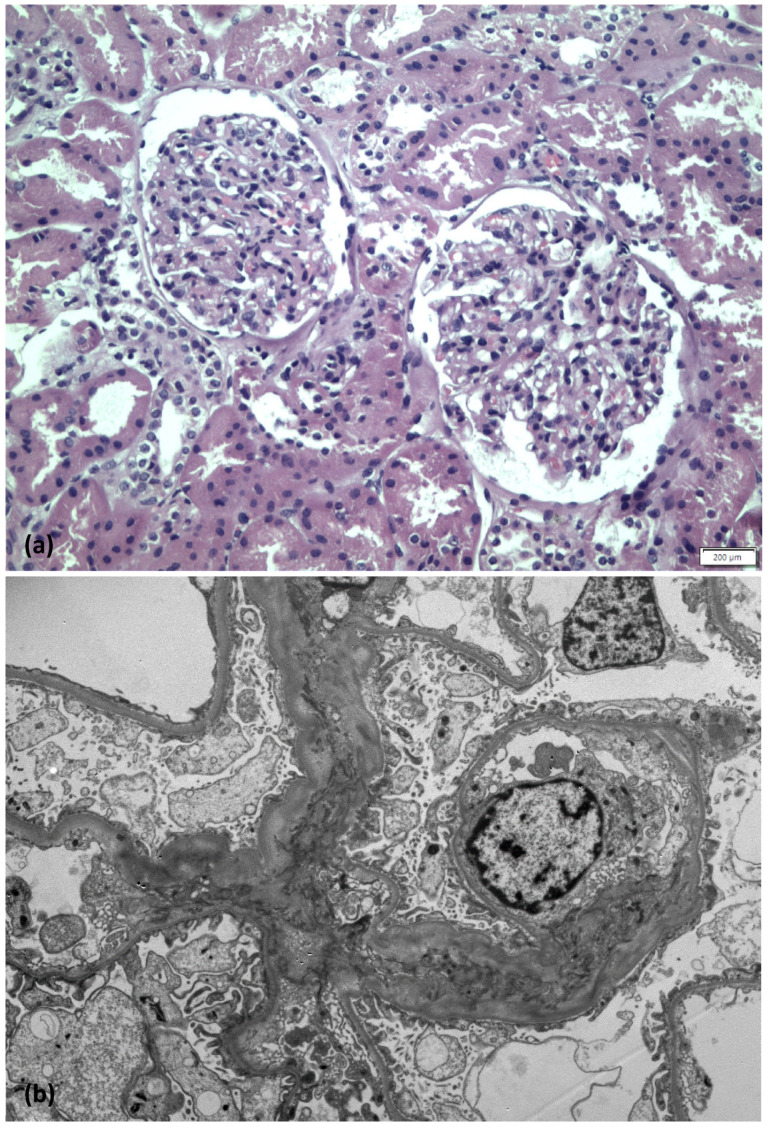
Images from light microscope (**a**), electron microscope (**b**).

On light microscopy, 25 glomeruli were identified—three of them with global sclerosis. Glomeruli appeared unremarkable; focally mild mesangial expansion was present. Tubular atrophy and interstitial fibrosis were mild, while vessels exhibited mild to moderate arteriosclerosis. Immunofluorescence examination revealed no immune deposits. Accordingly, on electron microscopy examination, no electron dense deposits were found, while diffuse foot process effacement with microvillous transformation was demonstrated (Figure 2). The final diagnosis was consistent with minimal change disease.

Oral prednisolone was promptly initiated at a dosage of 1 mg/kg daily and complete remission was achieved 10 days later.

## 3. Discussion

Herein, we report on a case of a living kidney donor, who developed de novo minimal change disease (MCD) 4 days after the 1st dose of the BNT162b2 mRNA COVID-19 vaccine (Pfizer/BioNTech).

Several publications reporting the relapse of a pre-existing or the development of a de novo glomerular disease after SARS-CoV-2 vaccination have emerged so far, as the literature about this rare entity is growing rapidly [1]. This unusual but severe “vaccine-related” or “vaccine-induced” complication is by no means new; cases of new-onset MCD and vasculitis after influenza vaccination have been previously reported [2].

To the best of our knowledge, a total of seven cases with de novo MCD post-SARS-CoV-2 vaccination had been described by the time this case report was written. Five cases arose after vaccination with the BNT162b2 mRNA (Pfizer/BioNTech)—one after the mRNA-1273 (Moderna) vaccine [1], and one after the adenovirus-vector ChAdOx1 (Astra-Zeneca) vaccine [3,4]. In most cases, MCD occurred shortly (4–14 days) after the first dose, presenting as abrupt onset of clinically overt nephrotic syndrome. The age spectrum in adults ranged from 19 to 82 years and the disease did not always follow a benign course, since four out of the seven cases were also complicated by acute kidney injury (AKI). All seven patients with biopsy-proven vaccine-related MCD received high-dose corticosteroid treatment. In six out of seven patients for whom outcomes were reported, either partial (2 out of 6) or complete (4 out of 6) remission occurred soon after treatment initiation.

Consistent with the other reported cases, MCD occurred early in our patient—4 days after the 1st vaccine dose. Due to the rapid spontaneous remission of the nephrotic syndrome, we decided that she could proceed with the 2nd dose, which eventually triggered the persistent full-blown nephrotic syndrome that forced us to intervene in terms of diagnosis and treatment.

A percutaneous, ultrasound-guided biopsy was performed on her remnant kidney. Though challenging, the intervention was uncomplicated, confirming the diagnosis of MCD. Therapy with oral high-dose corticosteroids (1 mg/kg/day) was initiated immediately and complete remission was achieved within 10 days. Most cases reported so far responded rapidly to steroid administration. A substantial proportion of described cases were complicated by AKI, which is not uncommon in adults with severe nephrotic syndrome, especially in the older population [5]. Our patient’s renal function remained normal throughout the disease course, despite her solitary kidney.

Though it is difficult to prove definitive causality, the potential relationship of de novo MCD with the SARS-CoV-2 vaccine is based on the close temporal association and the absence of other inciting factors. The pathophysiology involves a strong T-cell mediated response triggered by the spike protein, which enters the host through different mechanisms depending on the vaccine technology. The T-cell response induces the release of pro-inflammatory cytokines, such as TNF-α, IFN-γ, and IL-2, which eventually leads to podocytopathy similarly to that induced by the virus itself [6]. Notably, the most common glomerular lesion reported in SARS-CoV-2 infected individuals is collapsing focal segmental glomerulosclerosis (FSGS) [7].

Our case further contributes to the existing literature regarding vaccine-linked de novo glomerular diseases. Our case has two unique features. The first feature is the unlucky coincidence of this rare entity in a living kidney donor, which posed an additional challenge in terms of an urgent need for successful diagnosis and treatment. Fortunately, the diagnosis was quickly established, and complete remission was rapidly achieved. The second feature is the unusual disease course: nephrotic syndrome with a rapid, spontaneous remission shortly after the 1st vaccine dose and sustained, full-blown nephrotic syndrome with anasarca after the 2nd dose. This clinical presentation further strengthens the pathogenetic association between vaccination and de novo MCD. On the other hand, it raises the question of whether the second vaccine dose should have been postponed. Taking into consideration the lack of any reliable data, the rapid spontaneous remission after the first dose, and the strong indication for “cocooning”-protective vaccination for her immunocompromised husband in the middle of the 4th wave of the SARS-CoV-2 pandemic, we decided to proceed with the second dose. If more booster doses become advised for the general population, it remains challenging to decide if and with what vaccine platform technology; such severe complications developed by certain patients could be revaccinated.

## 4. Conclusions

Data about this rare entity are emerging and should be interpreted rigorously. Since millions of doses of vaccines are continuously administered worldwide and many cases may not be reported or published, a systematic registry recording de novo GN cases after SARS-CoV-2 vaccination would be a useful initiative.
